# *In silico* simulation of hyperoside, isoquercetin, quercetin, and quercitrin as potential antivirals against the pNP868R protein of African swine fever virus

**DOI:** 10.14202/vetworld.2024.171-178

**Published:** 2024-01-20

**Authors:** Putri Pandarangga, Yohanes T. R. M. R. Simarmata, Adi Berci Handayani Liu, Dwi Ari Fitri Haryati

**Affiliations:** 1 Department of Clinic, Reproduction, Pathology, and Nutrition, Faculty of Medicine and Veterinary Medicine, Universitas Nusa Cendana, Kupang, 85001, Indonesia; 2 Department of Chemistry, Faculty of Math and Science, Gadjah Mada University, Yogyakarta, 55281, Indonesia

**Keywords:** hyperoside, isoquercetin, molecular docking, pNP868R, quercetin, quercitrin

## Abstract

**Background and Aim:**

African swine fever (ASF) causes disease in pigs with up to 100% mortality rates. There is no effective vaccine to protect against it. This study aimed to perform *in silico* docking of ASF virus (ASFV) pNP868R protein with potential flavonoid ligands to identify ligands that interfere with mRNA cap formation.

**Materials and Methods:**

The ASFV pNP868R protein was tested with hyperoside, isoquercetin, quercetin, and quercitrin in this *in silico* simulation. ASFV pNP868R protein was extracted from the Research Collaboration for Structural Bioinformatics Protein Data Bank (RCSB PDB) database with PDB ID 7D8U (https://www.rcsb.org/structure/7D8U). Standard ligands were separated from proteins using UCSF Chimera 1.13. The standard ligand was redocked to protein using AutoDockTools 1.5.6 with the AutoDock4 method for validation. In the docking process, the grid box size was 40 × 40 × 40 Å^3^ with x, y, and z coordinates of 16.433, −43.826, and −9.496, respectively. The molecular docking process of the proposed ligand–protein complex can proceed if the standard ligand position is not significantly different from its original position in the viral protein’s pocket. The root mean square deviation (RMSD), root mean square fluctuation (RMSF), and radius of gyration (RoG) of the hyperoside with the lowest energy binding need to be analyzed with molecular dynamics using Groningen machine for chemical simulation 5.1.1.

**Results:**

Molecular docking and dynamic simulation revealed that hyperoside had the most stable and compact binding to the pNP868R protein. Hyperoside binds to the protein at the minimum energy of −9.07 KJ/mol. The RMSD, RMSF, and RoG values of 0.281 nm, 0.2 nm, and 2.175 nm, respectively, indicate the stability and compactness of this binding.

**Conclusion:**

Hyperoside is the most likely antiviral candidate to bind to the pNP868R protein *in silico*. Therefore, it is necessary to test whether this flavonoid can inhibit mRNA capping *in vitro* and elicit the host immune response against uncapped viral mRNA.

## Introduction

African swine fever (ASF) is caused by the ASF virus (ASFV) and is the most dangerous swine disease that causes up to 100% mortality. ASFV is a double-stranded DNA virus of the *Asfarviridae* family [1]. The ASFV genome ranges from 170 to 194 kbp with 150 open reading frames, of which almost half are of unknown function [2]. ASFV has 68 viral proteins with roles in viral structure (24%), viral transcription (19%), genome integrity (6%), entry into cells (4%), and avoiding host defense (3%), while 34% have no known function [3]. pNP868R is a genome integrity protein that helps cap RNA [4]. RNA caps can be a target for antivirals because imperfect RNA cap formation can interfere with viral growth and RNA can be recognized by the innate immune response [5].

There is no effective vaccine against this virus; therefore, identifying potential antivirals using *in silico* methods is an attractive option [6]. Flavonoids are the most frequently used plant-derived classes of antiviral agents that work through several mechanisms [7]. Flavonoids and their derivatives, such as myricetin and myricitrin, which inhibit ASFV protease [8]; genkwanin, which inhibits virus entry into cells [9]; and genistein, which disrupts DNA synthesis [10], have also been used as antivirals against ASFV. However, the ASFV pNP868R protein, which plays a role in capping RNA, has not been explored as a target protein for antivirals. Similar to other eukaryotic organisms and viruses, capping ASFV RNA plays a role in RNA stability and translation [11] by ensuring efficient pre-mRNA splicing, initiating mRNA translation, and preventing premature mRNA degradation [12, 13]. The pNP868R protein also has a guanylyltransferase (GTase) function that maintains the structure and biochemical characteristics of the methyltransferase domain [11]. If viral RNA is not capped, it will be recognized by cellular innate immunity through pathogen recognition receptors in host cells [14, 15].

More flavonoid derivatives, such as quercetin, isoquercetin, quercitrin, and hyperoside, with antiviral potential have recently been identified. Quercetin stimulates interferon (IFN) alpha and IFN-stimulated genes in pig foot-and-mouth disease infections [16]. Isoquercetin, quercitrin, and hyperoside can also inhibit the influenza virus in humans [17].

Moreover, *in silico* interactions between flavonoid compounds and ASFV proteins remain obscure. This study used molecular docking to determine *in silico* whether hyperoside, isoquercetin, quercetin, and quercitrin interact with pNP868R in a way that inhibits RNA capping.

## Materials and Methods

### Ethical approval

This study has not used experimental animals; therefore, ethical approval is unnecessary.

### Study period and location

This study was conducted from January to June 2023 at the Veterinary Medicine Program Study, Universitas Nusa Cendana, Indonesia, and the Chemistry Laboratory, Gadjah Mada University, Indonesia.

### Viral protein preparation

ASFV viral protein selection was performed by searching electronic databases such as Google Scholar and PubMed [18] with keywords including host defense, viral transcription, genome integrity, and the names of specific ASFV proteins. Fifty ASFV proteins were retrieved from the RCSB protein data bank (RCSB PDB; https://www.rcsb.org/). Proteins were filtered based on the availability of natural ligands, and the crystal structure of the selected protein was extracted from the PDB database. UCSF Chimera 1.13 [19] was used to separate the standard ligand, solvent molecule, and unnecessary regions that bind to the viral protein receptor. Proteins and standard ligands were saved in pdb format files.

### Geometry optimization of ligands

The three-dimensional structures of hyperoside, isoquercetin, quercetin, and quercitrin, as proposed ligands, were modeled and optimized using Gaussian 09W software (Informer Technology Inc. https://gaussian-09w.software.informer.com/9.0/) with density functional theory, Becke’s three-parameter hybrid functional, and Lee Yang Parr (B3LYP) hybrid function optimization method with a variation of 6-31G basis set and the polarization function (d, p). Geometry optimization and energy minimization processes were applied to obtain a molecule’s most stable structure and lowest energy. The optimized flavonoid compound structure was saved and converted to a Sybyl Mol2 file.

### Absorption, distribution, metabolism, excretion, and toxicity prediction

ADMET predictions of ligands used as antiviral candidates were analyzed using ADMETlab 2.0 (https://admetmesh.scbdd.com/), a web server based on predicting the pharmacokinetic properties of antiviral candidates [20].

### Molecular docking procedure

Standard ligands were redocked to the viral protein using AutoDockTools 1.5.6 software and the AutoDock4 method [21] for validation. AutoDock4 reads the standard ligand.pdb file and binds the viral protein. For the docking process, the grid box size was 40 × 40 × 40 Å^3^ with x, y, and z coordinates of 16.433, −43.826, and −9.496, respectively. If the position of the standard ligand from the redocking result is not significantly different from its original position in the viral protein’s pocket, then molecular docking of the proposed ligand–protein complex can proceed. Sylbyl Mol2 file was used for the proposed ligands. Following the completion of the docking study, the ligand docked pose was chosen based on the lowest binding affinity values. The molecular docking method is valid and has good resolution if the root mean square deviation (RMSD) value of redocking is <2.00 Å. The ligand–protein complex with the minimum binding energy can then be analyzed using molecular dynamics (MD) simulations [22].

### MD simulation

The Groningen MAchine for Chemical Simulation (GROMACS) software (https://www.gromacs.org/) was used to simulate protein interactions with selected ligands using the lowest binding affinity value [23]. The ligand and protein topologies were the initial input in the system simulation. In addition, GROMOS96 54a7 controlled the force field of the simulation system [24]. A cubic box with water as solvent was initially used to perform all the simulations. Ion addition and energy minimization were performed at 50 ns with a target temperature of 300 K, 1 atm. Subsequently, the ligand and protein complex were equilibrated using a system exposed to a solvent such as water. It was determined by specifying the number of particles (N), system volume (V), and temperature (T), that is, NVT equilibration, and the number of particles (N), system pressure (P), and temperature (T), that is, NPT equilibration. To achieve the best molecular docking results, the final setup for the MD simulation was run at 50 ns. The simulation results were analyzed using RMSD, RMSF, and RoG by specifying the system trajectory analysis in GROMACS as GMX RMSD, GMX RMSF, and GMX gyrate, respectively. The position, stability, and compactness of protein and ligand complexes in a dynamic system are measured in this analysis.

## Results

### Molecular docking prediction

**Figure-1:**
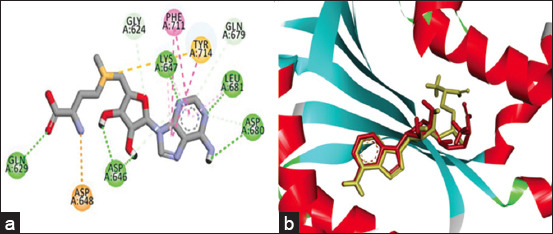
The interaction of standard ligand with pNP868R protein. (a) The binding of standard ligand to pNP868R protein after redocking using hydrogen (green dashes), π – π T-shaped (pink dashes), and π sulfur (yellow dashes) bindings. (b) The comparisons of standard ligand’s position before (green) and after (red) redocking process to pNP868R protein.

pNP868R was chosen for analysis based on the ligands and the crystal structures available in the PDB database. The standard ASFV pNP868R protein–ligand was redocked to the viral protein and produced a 1.89Å RMSD value. This value was <2Å, indicating that the structural conformation of the ligand did not change significantly after redocking. A standard ligand that binds to amino acid residues of the pNP868R protein required −3.41 kJ/mol as binding energy. Figure-1a depicts the binding between the standard ligand and the protein. This ligand formed six hydrogen bonds on amino acid residues, namely, Gln629, Asp646, Lys647, Leu681, and Asp680. Simultaneously, the standard ligand is connected to amino acid residues such as Gly624 and Gln679 by carbon-hydrogen binding. Other hydrophobic bindings, consisting of two π–π T-shaped bindings at Phe711 and Tyr714, were formed by the ligand. Moreover, π-sulfur binds to Tyr714 and Asp648 residue proteins.

The standard ligand–ASFV pNP868R protein interaction was stable, indicating that the standard ligand position did not significantly shift after redocking (Figure-1b). Thus, this approach can be applied to the flavonoid compound ligands proposed. Molecular docking determines the binding energy when the ligand is docked into the pNP868R protein. Based on the highest to the lowest energy requirements, the energy binding of the flavonoid compounds, namely, quercetin, isoquercetin, quercitrin, and hyeproside, was −7.65, −7.92, −8.5, and −9.07 KJ/mol, respectively. In general, ligand–protein complex interactions require hydrogen and hydrophobic bindings consisting of π-π T-shaped, π-cation, and π-alkyl bonds, respectively.

**Figure-2:**
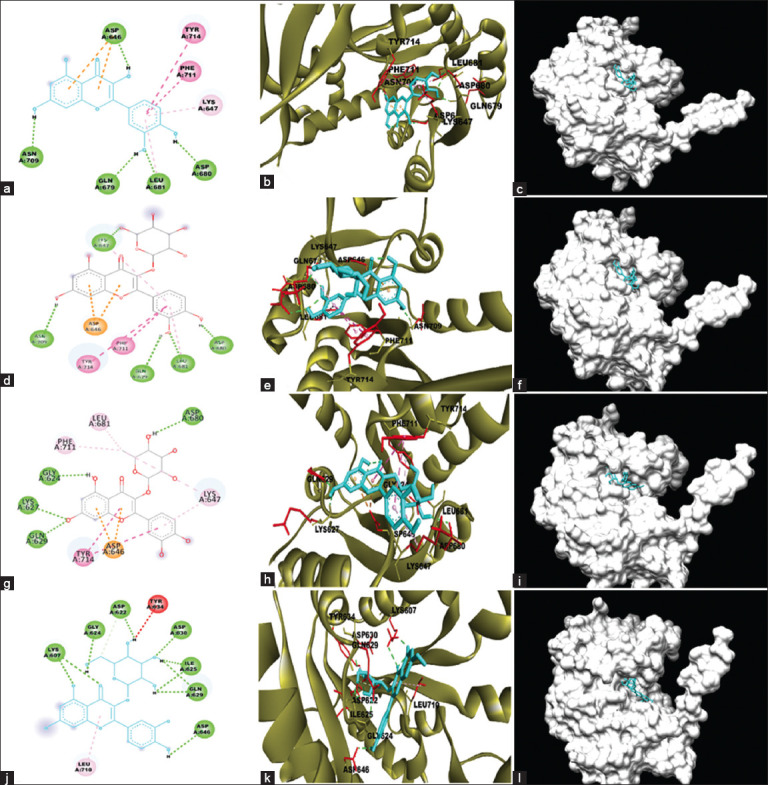
Interaction between flavonoid compounds with pNP868R protein shown in 2D and 3D images. (a) Quercetin binds to pNP868R protein using five hydrogen bindings and five hydrophobic bindings. (b) The interaction of quercetin (blue shape) with the receptor on the protein active site (green ribbons) connects with hydrogen and hydrophobic bindings. (c) The position of quercetin (blue shape) as a ligand in the pocket of viral protein (gray shape). (d) Isoquercetin interacted with pNP868R protein using five hydrogen and five hydrophobic bindings. (e) The interaction of isoquercetin (blue shape) with the receptors on the protein active site (green ribbons) that connect with hydrogen and hydrophobic bindings. (f) The position of isoquercetin (blue shape) as a ligand in the pocket of viral protein (gray shape). (g) Quercitrin binds to pNP868R protein using four hydrogen bindings and six hydrophobic bindings. (h) The interaction of quercitrin (blue shape) with the receptor on the protein active site (green ribbons) connects with hydrogen and hydrophobic bindings. (i) The position of quercitrin (blue shape) as a ligand in the pocket of viral protein (gray shape). (j) Hyperoside binds to pNP868R protein using ten hydrogen bindings and two hydrophobic bindings. (k) The interaction of hyperoside (blue shape) with the receptor on the protein active site (green ribbons) connects with hydrogen and hydrophobic bindings. (l) The position of hyperoside (blue shape) as a ligand in the pocket of viral protein (gray shape). The binding interactions: hydrogen bindings represented by green dashes; hydrophobic bindings consisting of π – π T-shaped binding represented by pink dashes; π – Alkyl binding represented by pastel pink dashes; π anion binding represented by orange dashes; and unfavorable donor binding represented by red dashes.

In molecular docking, quercetin binds to the active sites of the pNP868R protein with energy −7.65 KJ/mol. The interactions between quercetin and viral protein are depicted in Figures-2a–c. Asp646, Gln679, Asp680, Leu681, and Asn709 formed five hydrogen bindings between quercetin and active site residues. Hydrophobic interactions such as π-alkyl binding and two π-π T-shaped bindings with Lys647, Tyr714, and Phe711, respectively, were observed. Two π–π-anion bonds were connected at Asp646 for further hydrophobic binding. The ligand surfaces were predominantly exposed to water as a solvent rather than to amino acid residues (Figure-2b). When the ligand in the protein pocket was exposed to solvent in the 3D image (Figure-2c), some ligand surfaces were clearly visible.

Isoquercetin formed five hydrogen binding residues, Lys647, Asn709, Gln679, Asp680, and Leu681, at the active site residues (Figure-2d). The molecular docking predicted the binding energy to be −7.92 KJ/Mol. Isoquercetin formed two π-alkyl bindings at Lys647 and Leu681, which were hydrophobic. During hydrophobic binding, Isoquercetin formed a π–π T-shaped binding with Phe711 and Try714. Moreover, the two π-anion bindings with Asp646 were also formed by this ligand. Like quercetin, Figures-2e and f show that the isoquercetin surface is predominantly exposed to water as a solvent.

The predicted binding energy of the quercitrin ligand–protein complex was −8.5 KJ/mol. Quercitrin formed four hydrogen bonds at the active site residues of Gly624, Lys627, Gln629, and Asp680 (Figure-2g). Quercitrin also formed hydrophobic bindings, such as two π - π T-shaped bonds with Tyr714 and four π-alkyl bonds with Lys647, Leu681, and Phe711. Compared with quercetin and isoquercetin, the surface area of this ligand was dominant for attaching to amino acid proteins (Figures-2h and i).

Hyperoside required the smallest binding energy (−9.07 KJ/mol) compared to the other ligands. Hyperoside interacted with the active site of the protein by ten hydrogen bonds at Lys607 (twice), Asp622, Gly624, Ile625 (twice), Gln629 (twice), Asp630, and Asp646 (Figure-2j). This interaction also formed π-alkyl hydrophobic bindings with Leu710. In addition, the hyperoside formed an unfavorable donor binding at Tyr637. Based on the 3D images (Figures-2k and l), almost the entire ligand surface binds the protein receptors. In addition, hyperoside can be used as a candidate for further MD simulation analysis.

### MD simulations

MD simulation is a follow-up method of molecular docking that ensures the position, stability, and compactness of the ligand on the pNP868R protein residues. Hyperoside was chosen for further testing in MD simulation because it has the most negative binding affinity among the tested ligands. In this simulation, RMSD, RMSF, and the RoG were analyzed.

**Figure-3:**
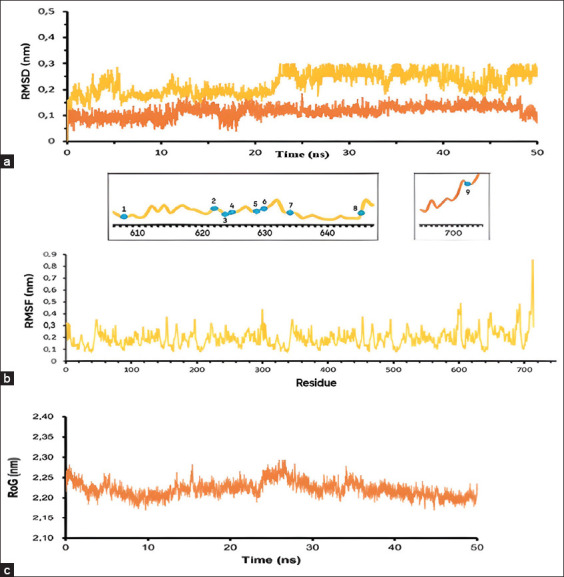
The position, stability, and compactness of hyperoside ligand when interacted with pNP868R using RMSD, RMSF, and RoG as trajectory systems in molecular dynamic analysis. (a) Comparison of the position movement of hyperoside ligand (yellow) and standard ligand (orange) when ligands in pNP868R protein pocket. The X-axis (time-ns) is the time requirement for the ligand to move the position, while the Y-axis (RMSD-nm) is the distance of the ligand shifts the position when tethered to the protein. (b) Stability of interaction between hyperoside and pNP868R protein at amino acid residues, namely 1. Lys607 (0.211 nm); 2. Asp622 (0.239 nm); 3. Gly624 (0.217 nm); 4. Ile625 (0.220 nm); 5. Gln629 (0.189 nm); 6. Asp630 (0.185 nm); 7. Tyr634 (0.195 nm); 8. Asp646 (0.239 nm); 9. Leu710 (0.283 nm). The X-axis shows the amino acid residues in the complex, while the Y-axis shows the fluctuation distance of the ligand toward the protein (nm). (c) Interaction compactness between hyperoside and pNP868R protein. The X-axis represents the distance (nm), while the Y-axis shows the time (ps). RMSD: Root mean square deviation, RMSF: Root mean square fluctuation, RoG: Radius of gyration.

The RMSD analysis is the first analysis performed at the MD simulation stage. The position deviation of the carbon atoms in a complex ligand compound was determined in a specific time frame. Figure-3a shows a comparison of the RMSD graph for the hyperoside ligand–protein complex (yellow) with the standard ligand–protein complex (orange). The ligand–protein complex fluctuated between 0 and 22 ns. However, it was stable between 23 and 50 ns. Despite the fluctuation during the specific time, the average RMSD value of 0.254 nm was still within the accepted value range. However, the fluctuations of the standard ligand–protein complex tended to be stable with an RMSD value of 0.115 nm. Compared to the standard ligand–protein complex, the hyperoside ligand movement toward the protein was stable and almost approached the standard ligand–protein movement pattern. However, if these fluctuations persist beyond the specified time (50 ns), these fluctuations will no longer be significant.

The stability value of the ligand–protein complex interaction was calculated using the RMSF property (Figure-3b). Hyperoside binds to nine amino acid residues of the pNP868R protein, that is, 1. Lys607 (0.211 nm); 2. Asp622 (0.239 nm); 3. Gly624 (0.217 nm); 4. Ile625 (0.220 nm); 5. Gln629 (0.189 nm); 6. Asp630 (0.185 nm); 7. Tyr634 (0.195 nm); 8. Asp646 (0.239 nm); and 9. Leu710 (0.283 nm). The average RMSF value was 0.219 nm, indicating the stability of the hyperoside–ASFV protein interaction.

RoG analysis was performed to determine the compactness of the complex by looking at the spectrum formed in the graph (Figure-3c). The highest and lowest RoG values were observed at 2.3 and 2.1 nm. However, the average RoG value is 2.175 nm. The RoG value shows that the binding between the hyperoside ligand and the protein was compact, indicating that hyperoside is an antiviral candidate for ASFV.

## Discussion

Among the four flavonoid compounds proposed as ASFV antiviral candidates targeting the pNP868R protein, only hyperoside was selected. Based on molecular docking results, we selected hyperoside, which required a minimum energy of −9.07 KJ/mol. The smaller the energy required by the ligand to interact with the viral protein, the better it is to be used as an antiviral candidate. We applied MD to confirm hyperoside as an antiviral candidate for the pNP868R protein from ASFV.

MD aims to determine the position movement, stability, and compactness of hyperoside when it interacts with the pNP868R protein. The RMSD value indicates that the movement of the hyperoside does not shift too much compared with that of the standard ligand when interacting with the pNP868R protein. Therefore, hyperoside can replace the standard position of the pNP868R ligand. Hyperoside as an antiviral agent can also be determined using the RMSF value to show the stability of hyperoside when interacting with amino acid residues on the pNP868R protein. The average RMSF value was 0.2 nm. This value indicates that the hyperoside ligand–protein complex bond is stable. Moreover, the position of the amino acid residue for the hyperoside attachment site was like that of the standard ligand towards the pNP868R protein. Gly624, Gln629, and Asp646 have acceptable RMSF values of 0.217 nm, 0.189 nm, and 0.239, respectively. The compactness of the hyperoside ligand and viral protein binding can be seen in the RoG value of 2.175 nm. The results of molecular docking and MD showed that hyperoside was stable in replacing the standard ligand of the pNP868R protein. Therefore, it can be used as an antiviral candidate.

Based on ADMET results, hyperoside violates Lipinski’s five rules. However, these five rules are applicable only to oral drugs and cannot be applied to drug candidates derived from natural products [25]. Moreover, hyperoside has poor bioavailability when administered orally; therefore, intervascular and intraperitoneal administration is more effective [26]. Therefore, Lipinski’s five rules do not apply to hyperoside.

There has been no effective vaccine against ASFV [27]. Therefore, antiviral drugs such as genistein, which inhibit viral transcription from ASFV [10], have begun to be searched for ASF. The ASFV produces more than 150 proteins. However, only a few antiviral drugs have been tested against ASFV using protein-protein interaction analysis [28]. Many other important proteins, such as the pNP868R protein, have not yet been explored regarding immune response stimulation. This protein is controlled by the NP868R gene, which is involved in GTase activity in the formation of mRNA capping [11].

The functions of mRNA capping include stability, protein translation, and concealment from cellular proteins that recognize foreign RNA [12]. Capping RNA can prevent retinoic acid-inducible gene I (RIG-I) from entering host cells, thereby inducing immunity [29]. To avoid the immune response is a defense mechanism for the virus so that it continues to replicate in host cells. ASFV mRNA will be recognized by RIG-I with the presence of hyperoside as a ligand to inhibit the action of the pNP868R protein in making mRNA capping. Thus, this early detection will activate pro-inflammatory cytokines such as tumor necrosis factor (TNF) and IFN through the nuclear factor-κB (NF-κB) transcription factor. However, excessive production of cytokines by the host has destructive effects on the cells, especially endothelial cell dysfunction [30]. The consequence is bleeding.

Despite the presence of an mRNA cap, the ASFV can evade the immune response by suppressing the interferon regulatory transcription factor 3, so it does not produce IFN as a strong antiviral [31]. In contrast, this virus also stimulates pro-inflammatory cytokines that can damage endothelial cells. As a result, heavy bleeding almost throughout the pig’s body [32] is characteristic of this disease. Moreover, the virus stimulates macrophages to release nitric oxide and vascular endothelial growth factor B to increase permeability, thereby stimulating capillary leakage and diapedesis hemorrhage [33]. Hyperoside can act as vasoprotective by reducing the levels of NF-κB, TNF receptor 1, and extracellular-signal-regulated kinase, thus inhibiting excessive inflammation in the vasculature [34]. In addition, hyperoside can inhibit vascular permeability via the acetic acid-induced mechanism [35] when excessive cytokine release due to ASF causes vascular permeability of the host.

Thus, we speculate that hyperoside may protect blood vessels in pigs from cytokine attacks caused by the ASFV, apart from inhibiting the action of capping mRNA formation by pNP868R protein. However, further investigation is needed to understand how hyperoside works as a specific antiviral against ASFV, which has several proteins that stimulate the immune response of pigs in complex ways. In addition, hyperoside has many benefits, such as anticancer, neuroprotective, cardioprotective, hepatoprotective, brain-protective, lung-protective, and vasoprotective, as summarized by Xu *et al*. [26].

## Limitations

Hyperoside investigations have been conducted using *in silico* methods, the results of which will vary if used directly *in vivo* due to the complexity of the ligand–protein complex. Based on the results of molecular docking, MD simulation, and previous study data, hyperoside is a good candidate as an ASF antiviral that inhibits pNP868R protein. However, long-term use of hyperoside can accumulate in the body, especially in the kidneys. Therefore, toxicity, range time, and dosage of hyperoside should be tested. In this study, we focused only on pNP868R protein, whereas ASFV has more than 150 proteins that work in a complex manner. Analyzing several ASFV proteins related to the host immune response is also necessary to gain a more comprehensive understanding of the effectiveness of hyperoside.

## Conclusion

Based on the docking results, hyperoside was selected as an antiviral candidate for pNP868R proteins. The position, stability, and compactness of hyperoside toward pNP868R protein were shown using MD depicted with RMSD, RMSF, and RoG values of 0.281 nm, 0.2 nm, and 2.175 nm. This binding inhibits the work of the pNP868R protein to form mRNA capping for ASFV so that it can be recognized directly by the host receptor to stimulate innate immunity, particularly antiviral immunity.

## Authors’ Contributions

PP, YTRMRS, ABHL, and DAFH: Designed the study and edited the manuscript. PP: Drafted the manuscript. ABHL and DAFH: Performed the molecular docking and molecular dynamics. PP, ABHL, and DAFH: Performed the data analysis. All authors have read, reviewed, and approved the final manuscript.
